# Commentary: Assessing MRI interpretability of the orbit, paranasal sinuses, and nasopharynx in cochlear implant patients

**DOI:** 10.3389/fneur.2025.1691857

**Published:** 2025-11-13

**Authors:** Memik Teke

**Affiliations:** Department of Radiology, University of Colorado Anschutz Medical Campus, Aurora, CO, United States

**Keywords:** MRI, diffusion weighted imaging (DWI), cochlear implants, orbit, paranasal sinuses

## Introduction

Ketterer et al. (1) address an important practical question: how interpretable are MRI sequences of the orbit, paranasal sinuses, and nasopharynx in patients with cochlear implants (CIs)? The topic has clear clinical relevance given artifact susceptibility and safety considerations in CI carriers. While the intent is commendable, several methodological and interpretative issues limit the reliability and generalizability of the conclusions.

## Methodological limitations: sample and model

### Single-participant design

The study was performed on a single healthy volunteer. The authors have clearly acknowledged this limitation, noting that their work was exploratory in nature. While this design provides valuable preliminary insights under controlled conditions, it inherently limits external validity and the ability to capture interindividual variability. Future studies including multiple subjects with diverse implant models and anatomical differences could build upon these findings and strengthen generalizability.

### External fixation instead of surgical implantation

The CI device was externally fixed with a headband rather than surgically implanted. This configuration does not reproduce the *in vivo* interface with bone and soft tissues, key determinants of magnetic susceptibility behavior and artifact propagation, especially on sequences such as DWI, ADC, and SWI (2). As a result, the reported image quality and sequence “feasibility” may not reflect real-world conditions.

### Ethical reporting inconsistency

The manuscript contains an apparent inconsistency: early on it states that informed consent was obtained from the participant, whereas the ethics section later indicates that written consent was waived due to a retrospective design. Given that the imaging protocol was prospectively applied to a living volunteer, this discrepancy raises concerns about the clarity of the study classification and the accuracy of ethics reporting. The authors should reconcile these statements and specify the IRB/ethics pathway that governed the prospective procedures.

### Text–figure discrepancy and interpretational overreach

There is a noticeable contradiction between the conclusions presented in the main text and the data shown in the figures. The authors claim: “It was observed that orbital MRI diagnostics in the required sequences (T1, T2, and DWI) are feasible even in patients with bilateral CIs with magnets *in situ*.” However, Figure 2 states: “In DWI, orbital accessibility for 135° cochlear implant positioning was impeded with the magnet in place unilaterally (a) or bilaterally (c), whereas without the magnet (b), the orbit was clearly visible and accessible.”

## Discussion

In conclusion, although the intention to improve MRI protocols for CI patients is commendable, the study's methodological limitations, inconsistencies in ethical reporting, and internal contradictions between textual claims and figure content reduce its scientific robustness and clinical relevance.
